# Concomitant and decoupled effects of cigarette smoke and SCAL1 upregulation on oncogenic phenotypes and ROS detoxification in lung adenocarcinoma cells

**DOI:** 10.1038/s41598-021-97869-1

**Published:** 2021-09-15

**Authors:** Carmela Rieline V. Cruz, Jose Lorenzo M. Ferrer, Reynaldo L. Garcia

**Affiliations:** 1 Disease Molecular Biology and Epigenetics Laboratory, National Institute of Molecular Biology and Biotechnology, University of the Philippines Diliman, 1101 Quezon City, Philippines; 2 Department of Developmental Biology, International Max Planck Research School, Justus-von-Liebig-Weg 11, 37077 Göttingen, Germany

**Keywords:** Cancer, Molecular biology, Molecular medicine, Oncology, Pathogenesis

## Abstract

Lung cancer is the leading cause of cancer deaths worldwide, with smoking as its primary predisposing factor. Although carcinogens in cigarettes are known to cause oncogenic DNA alterations, analyses of patient cohorts revealed heterogeneous genetic aberrations with no clear driver mutations. The contribution of noncoding RNAs (ncRNAs) in the pathogenesis of lung cancer has since been demonstrated. Their dysregulation has been linked to cancer initiation and progression. A novel long noncoding RNA (lncRNA) called smoke and cancer-associated lncRNA 1 (SCAL1) was recently found upregulated in smoke-exposed adenocarcinomic alveolar epithelial cells. The present study characterized the phenotypic consequences of SCAL1 overexpression and knockdown using A549 cells as model system, with or without prior exposure to cigarette smoke extract (CSE). Increase in SCAL1 levels either by CSE treatment or SCAL1 overexpression led to increased cell migration, extensive cytoskeletal remodeling, and resistance to apoptosis. Further, SCAL1 levels were negatively correlated with intracellular levels of reactive oxygen species (ROS). In contrast, SCAL1 knockdown showed converse results for these assays. These results confirm the oncogenic function of SCAL1 and its role as a CSE-activated lncRNA that mediates ROS detoxification in A549 cells, thereby allowing them to develop resistance to and survive smoke-induced toxicity.

## Introduction

Smoking is the number one predisposing factor for lung oncogenesis^1^. A significant proportion of lung cancer deaths is attributable to smoking—around 80% of patients in the United States and France, 61% of patients in Asia, and 40% of patients in sub-Saharan Africa^2^. Genome-wide analyses of expression patterns in adenocarcinomas reveal divergence in both genomic and transcriptomic landscapes between tumors that arise in smokers and never-smokers. In general, tumors obtained from never-smokers are characteristically less aggressive than those found in smokers^1^.

Although smoking is established as a driver of lung oncogenesis, molecular mechanisms that ultimately lead to an aggressive neoplastic phenotype remain unclear. It has been hypothesized that chronic exposure of pulmonary DNA to metabolically active carcinogens present in cigarettes result in DNA adducts and genetic lesions that may lead to carcinogenesis^3^. Although genetic alterations brought about by smoke-induced injury may be the cause of lung carcinomas, the mutations are heterogeneous across patient cohorts and it is difficult to identify driver mutations that are responsible for disease onset. Further, mutations in oncogenes and tumor suppressors may not be solely responsible for cancer of the lung. In recent years, non-coding RNAs including microRNAs, lncRNAs and circular RNAs, have been implicated in many cancer types. In lung cancer tissues, the cancer-associated region long noncoding RNA 5 (CARLo5)^4^, Differentiation antagonizing non-protein coding RNA (DANCR)^5, 6^, antisense non-coding RNA in the INK4 locus (ANRIL)^7^, lncRNA H19^8^, and colon cancer-associated transcript 2 (CCAT2)^9^, were found to be upregulated; while the BRAF-activated non-coding RNA (BANCR)^10^, maternally expressed gene 3 (MEG3)^11^, and taurine upregulated gene1 (TUG1)^12^ were shown to be downregulated. More recently, a study by Thai and colleagues revealed highly elevated levels of an lncRNA that was later named smoke and cancer-associated lncRNA 1 (SCAL1) in airway epithelial cells of smokers, which was also upregulated in lung cancer cells exposed to cigarette smoke^13^. Its discovery led to several hypotheses that suggest SCAL1 may translate the chemical cues of smoke exposure into concrete oncogenic stimuli such as epigenetic changes. The role of SCAL1 in oncogenesis now appears to be more universal and extends to other cellular contexts. It has been shown to promote proliferation and invasion in clear cell renal cell carcinoma^14^, hepatocellular carcinoma^15^, gastric cancer^16^, cervical cancer^17^, and glioma cells^18^; proliferation, invasion, cell cycle progression, and cell survival in papillary thyroid cancer^19^; proliferation, cell survival, metastasis and inhibition of tumor suppressor genes through DNA methylation in esophageal squamous cell carcinoma^20^; proliferation and induction of stem-like properties in breast cancer cells^21^; proliferation and survival in ovarian cancer^22^; and inhibition of apoptosis in colorectal cancer cells^23^.

SCAL1 is an 890-bp lncRNA that has been posited to initiate repair in epithelial cells after smoke-induced injury. In lung cancer cells, the expression of this lncRNA is necessary for combating DNA double-stranded breaks induced by chemotherapeutic agents such as cisplatin and doxorubicin^24^. SCAL1 expression is governed by nuclear factor erythroid 2-related factor 2 (NRF2), a transcription factor considered the master regulator of antioxidant genes, which normally protects cells from injury as a result of oxidative stress. Under physiological conditions, NRF2 is ubiquitinated and degraded in the cytosol due to its interaction with Kelch-like ECH-associated protein 1 (KEAP1). However, ROS generated upon electrophilic insult cause structural alterations in the KEAP1 protein, which in turn allows NRF2 to escape degradation and translocate into the nucleus to activate the expression of antioxidant genes. NRF2 can bind a putative nuclear factor-erythroid-2 (NFE2) binding site in the SCAL1 promoter, thus upregulating its expression. Moreover, analysis of publicly available transcriptome sequencing data of a large cohort of lung adenocarcinoma and squamous cell carcinoma revealed that SCAL1 expression correlated with KEAP1 and NFE2L2 (otherwise known as NRF2) mutational status^25^.

Although postulated to rescue lung cancer cells from the toxic effects of cigarette smoke exposure as shown in cell viability assays, the oncogenic effects of SCAL1 upregulation, either secondary to or decoupled from cigarette smoke, have not been elucidated. In this study, the phenotypic consequences of exposure to cigarette smoke extract (CSE) as well as SCAL1 overexpression and knockdown were investigated by studying their effects on migratory capacity, formation of invasive structures secondary to extensive cytoskeletal reorganization, resistance to apoptosis, and levels of reactive oxygen species (ROS).

## Results

### SCAL1 is upregulated in CSE-treated A549 cells

**Figure 1:**
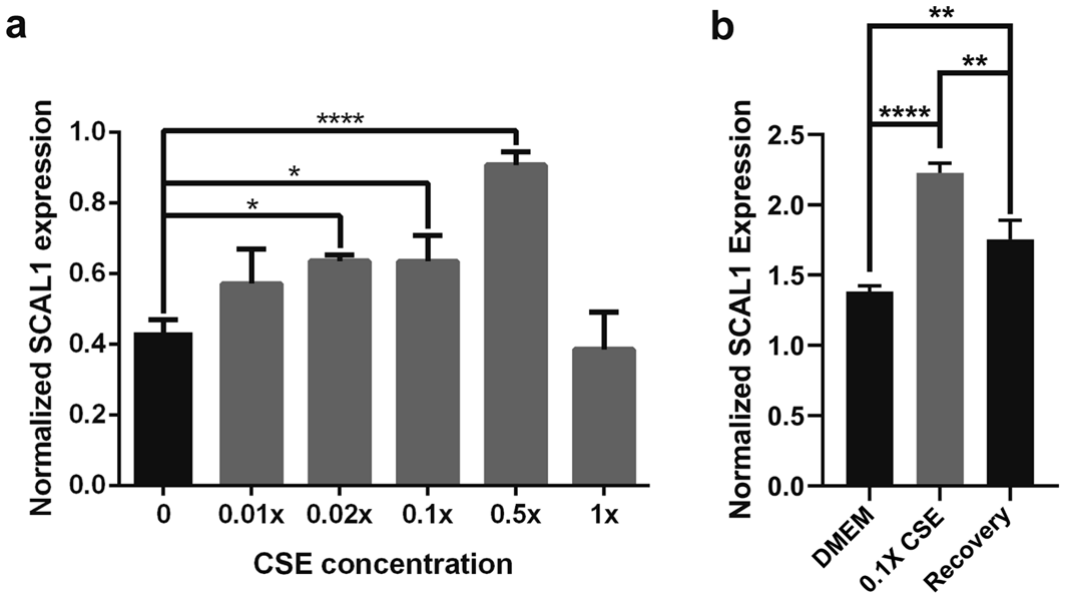
SCAL1 expression levels in CSE-treated A549 cells. (**a**) SCAL1 expression levels increased with increasing concentrations of cigarette smoke extract (CSE), but decreased when cultured at the highest concentration of CSE. (**b**) When cells were allowed to recover for 24 h in DMEM after 48 h of exposure to 0.1 × CSE, SCAL1 levels were significantly lower than if kept in 0.1 × CSE but still remained significantly higher than in the control setup. Data presented are representative of three independent trials and are expressed as the mean ± SD. *P < 0.05, **P < 0.01, ***P < 0.001 and ****P < 0.0001.

A549 adenocarcinomic alveolar epithelial cells were cultured in arbitrary dilutions of CSE-DMEM (0.01×, 0.02×, 0.1×, 0.5× CSE dilutions, and concentrated or 1× CSE) to facilitate dose-dependent studies on CSE-induced upregulation of SCAL1. In order to gauge SCAL1 lncRNA expression levels, total RNA was extracted from CSE-treated A549 cells after an incubation period of 48 h. SCAL1 levels were then compared across setups exposed to varying CSE concentrations. As shown in Fig. 1a, exposure to increasing CSE concentrations up to the twofold dilution setup was accompanied by a corresponding increase in the relative expression levels of SCAL1. However, a reduction of SCAL1 levels was observed in the concentrated CSE setup. SCAL1 expression levels were not significantly different from the levels seen in the untreated setup.

To determine if reverting CSE-exposed cells to CSE-free medium will affect SCAL1 expression, two setups of A549 cells were initially treated with 0.1× CSE. After 48 h, one setup was replenished with fresh 0.1× CSE medium while the other was switched to CSE-free medium. RT-qPCR analysis showed that SCAL1 expression went down after switching CSE-exposed cells to CSE-free medium (Fig. 1b) indicating that acute exposure followed by a recovery period can downregulate SCAL1 expression.

### SCAL1 overexpression phenocopies effects of cigarette smoke exposure on migration rate of A549 cells

Effects of SCAL1 lncRNA on the migratory capacity of A549 lung cancer cells were investigated via the scratch wound assay. Migration of cells was measured in CSE-treated, SCAL1 overexpression, and SCAL1 knockdown setups. Preliminary experiments showing overexpression and knockdown of SCAL1 in transfected cells are shown in Fig. S1.

**Figure 2:**
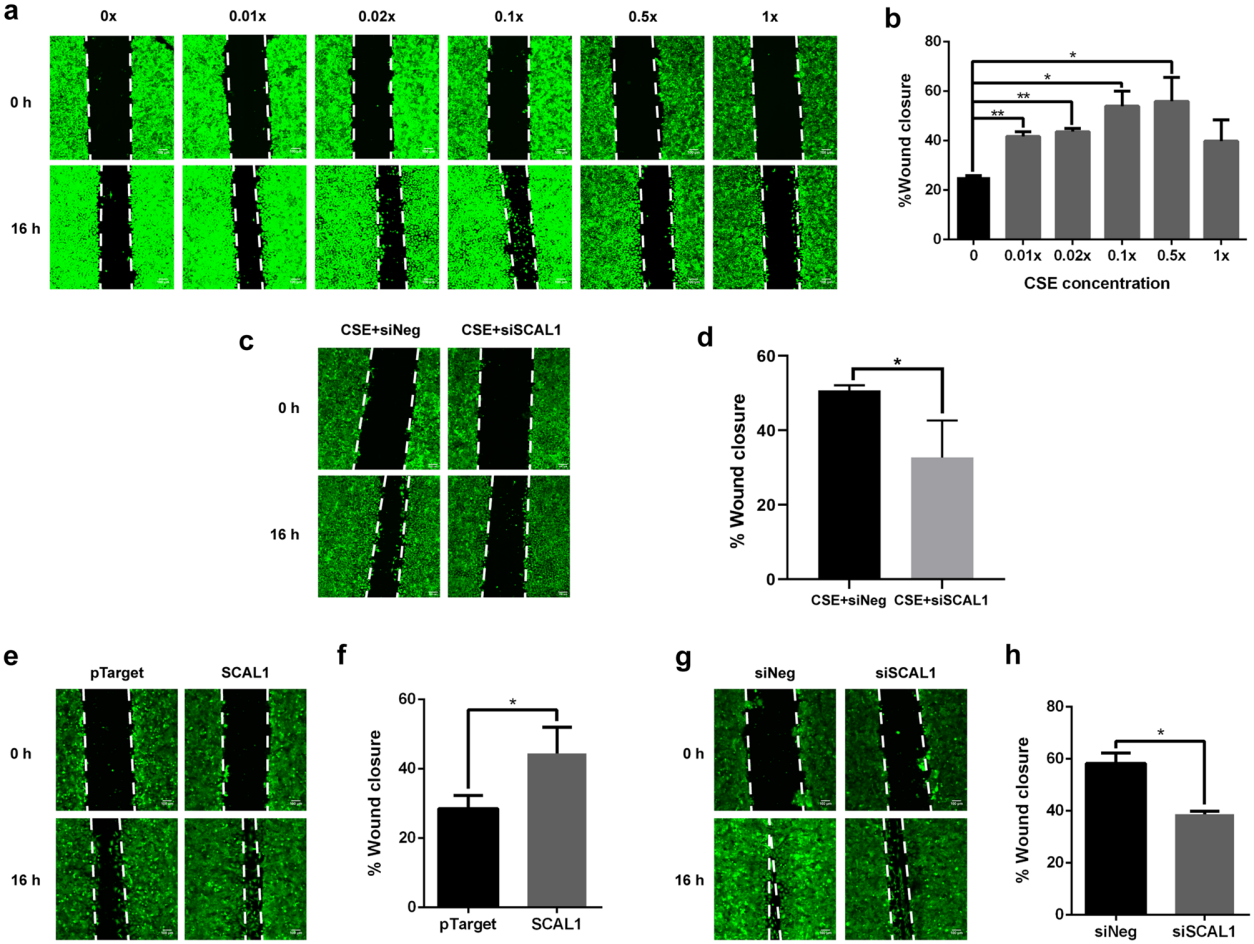
Scratch wound assay of CSE-treated A549 cells to measure migratory capacity. (**a**,**b**) Wound closure rates increased until the 0.5 × dilution setup but a sudden decrease was observed in the concentrated 1 × setup. (**c**,**d**) When SCAL1 was knocked down 24 h post-exposure to 0.1 × CSE, wound closure after 16 h was significantly reduced compared to the control. (**e**,**f**) SCAL1 overexpression increased wound healing rates while the converse was observed upon (**g**,**h**) SCAL1 knockdown. Images shown are representative of three independent trials done in triplicates. Data presented are representative of three independent trials and are expressed as the mean ± SD. *P < 0.05, **P < 0.01, ***P < 0.001 and ****P < 0.0001.

Migration rates of A549 cells exposed to different concentrations of CSE exhibited a similar trend as the RT-qPCR results of SCAL1 expression in Fig. 1a. Migration rates increased until the twofold dilution setup then decreased upon exposure to higher CSE levels (Fig. 2a,b). The increase in migratory capacity of A549 cells exposed to increasing concentrations of CSE, and the concomitant increase in the expression levels of SCAL1 suggest that the lncRNA may mediate the effects of cigarette smoke exposure, at least partially, in promoting cellular migration. To determine if indeed SCAL1 mediates the pro-migratory effect of CSE exposure, at least partially, A549 cells were treated with 0.1× CSE for 24 h to upregulate SCAL1 expression. The cells were then transfected with 30 nM siSCAL1 to knock down SCAL1 expression. Scratch wound assay confirmed that knockdown of SCAL1 in CSE-exposed cells significantly decreased migratory capacity of A549 cells (Fig. 2c,d).

Overexpression of SCAL1 in A549 cells phenocopied the effects of CSE exposure, and increased migration rate compared to the pTargeT™ empty vector control (Fig. 2e,f). SCAL1 knockdown, on the other hand, decreased migratory capacity of A549 cells compared to the siNeg (negative control) setup (Fig. 2g,h). Taken together, these experiments done in CSE-free medium suggest that although SCAL1 upregulation may partially mediate the pro-migratory effect of CSE, its effect on cellular migration can still be decoupled from that of CSE.

### Distinct and overlapping effects of CSE treatment and SCAL1 upregulation on cytoskeletal remodeling and formation of invasive structures

**Figure 3:**
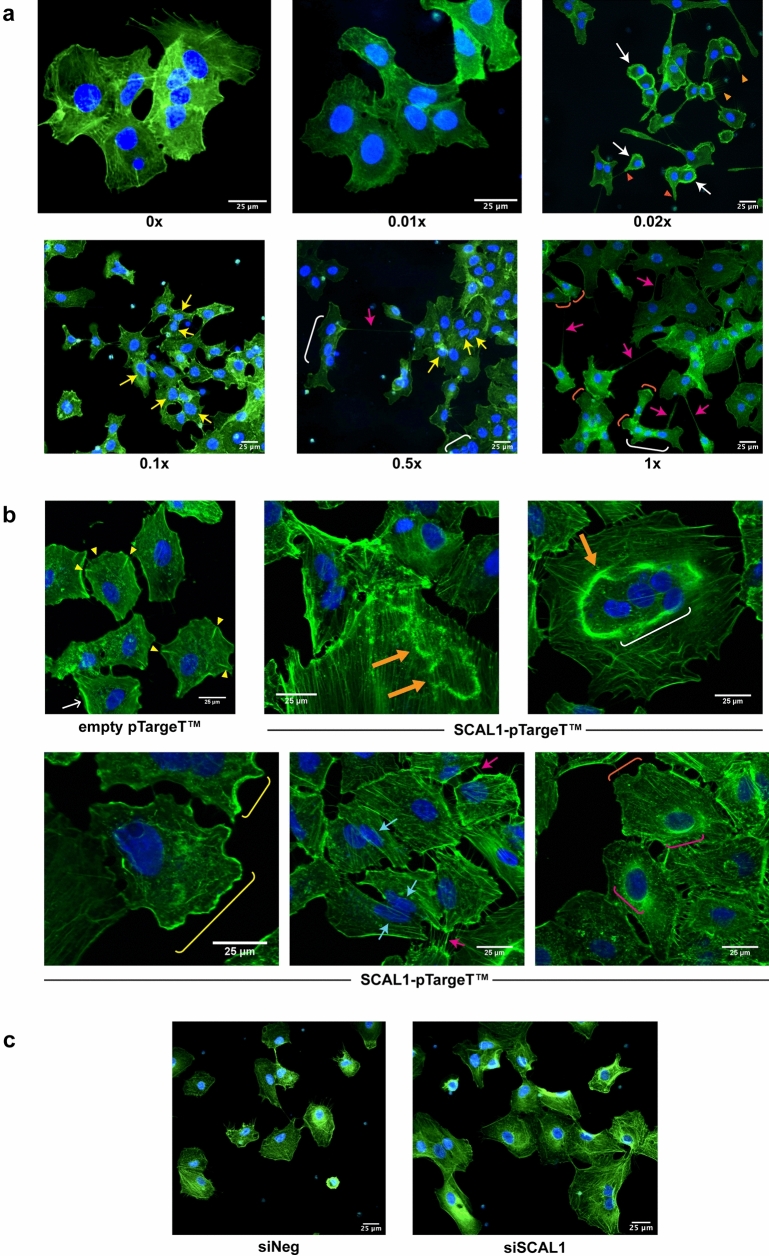
Fluorescent staining of CSE-treated, SCAL1-overexpressing and SCAL1-deficient A549 cells with Phalloidin-FITC and Hoechst 33342 (Olympus IX3-HOS, ×400, FITC: 500 ms). (**a**) A549 cells exposed to different CSE dilutions showed formation of structures associated with migration and invasion such as invadopodia (red arrowheads), hair-like filopodia (orange arrowheads), thick peripheral actin (white arrows), tunneling nanotube (TNT)-like structures (fuchsia arrows), multilobulated nuclei (yellow arrows), multinucleated cells (white brackets), and lamellipodia (orange brackets). (**b**) Empty vector-transfected control cells showed a stable cytoskeleton with prominent radial protrusions/focal adhesions (yellow arrowheads). Cells transfected with SCAL1 displayed transient structures such as circular dorsal ruffles (thick yellow orange arrows), peripheral dorsal ruffles (yellow brackets), stretched nuclei (cyan arrows) and perinuclear actin rims (fuchsia brackets). (**c**) A549 cells transfected with SCAL1-siRNA were more detached from one another and exhibited a more organized cytoskeleton with prominent stress fibers compared to siNeg controls.

To investigate the effects of CSE exposure and SCAL1 transcript levels on the invasive phenotype of A549 cells, the cytoskeletal architecture of treated or transfected cells was evaluated by phalloidin staining and fluorescence microscopy. Analysis of the cytoskeletal organization of CSE-treated cells revealed variable gross morphological changes compared to untreated control A549 cells. As shown in Fig. 3a, at 0× and 0.01× CSE, A549 cells were relatively large with prominent focal adhesions and stress fibers. Moreover, being an epithelial cell line, cell-to-cell adhesion facilitated by intercellular junctions were prominent. At higher CSE concentrations, A549 cells were observed to decrease interactions with neighboring cells. In 0.02× CSE, cytoskeletal reorganization was apparent with many cells assuming a round morphology with thick peripheral actin rim and nuclei against one side of the cell. Peripheral actin bundles are a positive indicator for lamellipodial formation and motility, and may also play a role in nuclear deformability during migration^26–28^. Filopodia, thin cellular protrusions implicated in metastatic spread, are identifiable in control untreated cells but more abundant in cells exposed to 0.02× CSE. Protrusive actin structures are also prominent. In 0.1× and 0.5× CSE, there is a preponderance of misshapen or multilobulated nuclei as well as those exhibiting blebs. Fused multinucleated cells are also more observable. Finally, cells exposed to higher concentrations of CSE (0.5× and 1×) developed more intercellular connections that resemble tunneling nanotubes (TNTs). TNTs are thin, actin-based intercellular bridges that allow the passage of organelles, plasma membrane components, and cytoplasmic molecules between connected cells and may further promote invasiveness^29–31^. Detached cells with cone-shaped migrating front and prominent lamellipodia, as well as multinucleated cells, can also be observed.

Overexpression of SCAL1 induced cytoskeletal changes that only partially overlap with those observed in CSE-treated A549 cells (Fig. 3b). Control cells transfected with pTargeT empty vector only were generally polygonal in shape with identifiable radial protrusions that represent stable focal adhesions which are able to prevent migration. Cells transfected with SCAL1 show more transient structures that can disrupt stress fibers, and promote or facilitate cellular migration. These include peripheral ruffles, circular dorsal ruffles (CDRs), perinucelar actin rims, and peripherally located stretched nuclei. Large multinucleated cells are also more observable.

In contrast, reduction of SCAL1 levels through RNA interference, led to relative stabilization of cytoskeletal architecture. As shown in Fig. 3c, cells transfected with SCAL1-specific siRNA exhibited a more organized actin cortex with parallel stress fibers and very few, shorter filopodia. Further, cells appear to be more adhered to each other. The nuclei are also more regular in shape. The siNEG control, on the other hand, showed loosely packed and criss-crossing actin filaments as well as more extensive filopodial formation, and are more solitary.

Overall, the distinct and overlapping effects of CSE treatment and SCAL1 upregulation on cytoskeletal reorganization suggest that although SCAL1 upregulation may partially mediate the effects of CSE, its effects on oncogenic phenotypes can still be decoupled from that of CSE.

### Cigarette smoke and SCAL1 promote resistance to apoptosis in A549 cells

**Figure 4:**
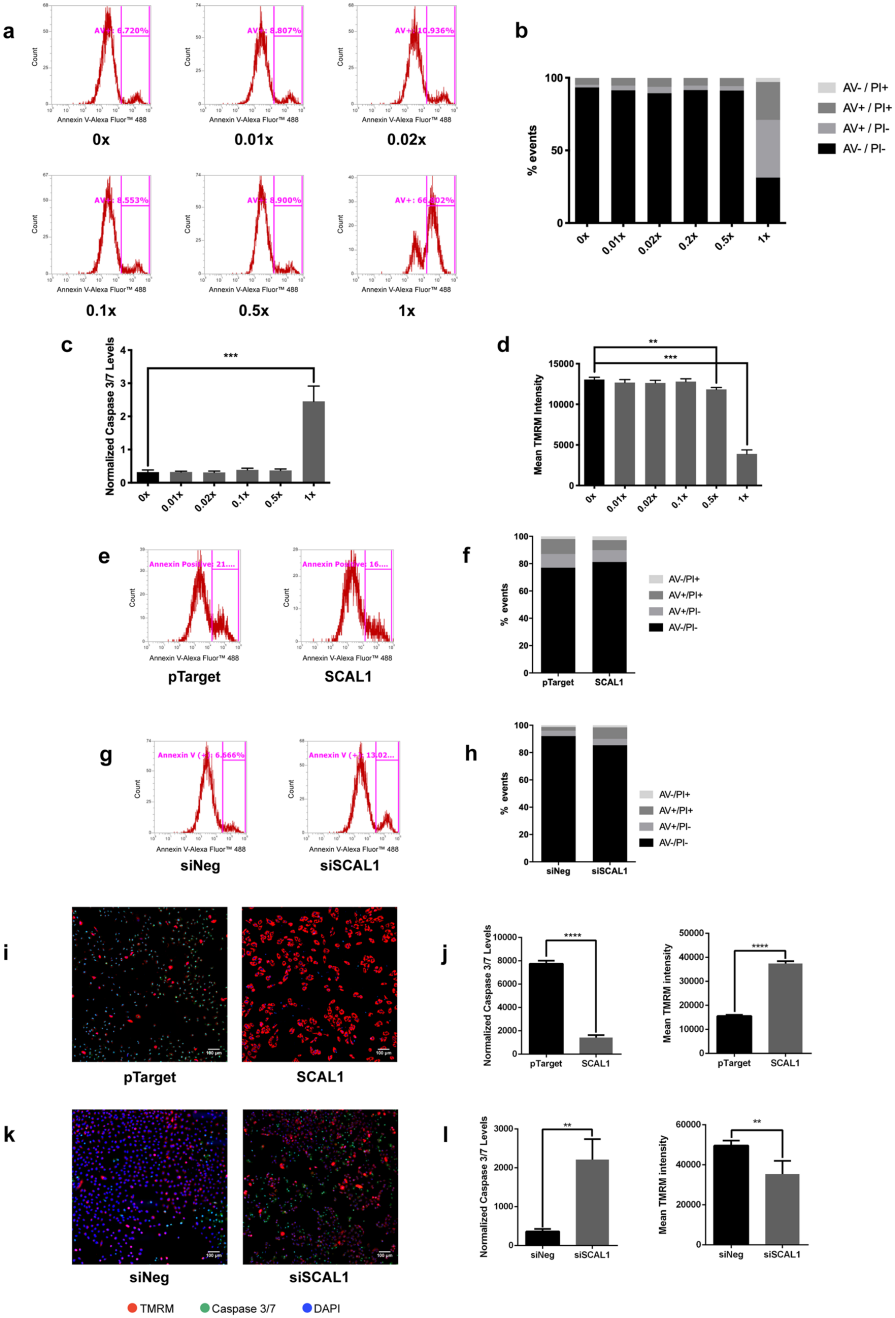
CSE treatment and SCAL1 expression enables A549 cells to resist apoptotic induction with MSB. (**a**) Annexin V-Alexa Fluor 488-gated events representing apoptotic cells were unaffected by CSE treatment, except for cells treated with 1× CSE. (**b**) Live cell populations (AV−/PI−) remained unchanged across different CSE treatments, except for the 1× CSE setup exhibiting a shift to more apoptotic (AV+) cells. (**c**) Low caspase 3/7 levels persisted in different CSE treatments but increased in the 1× CSE setup. (**d**) Mitochondrial integrity measured with TMRM intensity was unaffected by incubation with lower levels CSE but was greatly reduced in cells treated with 1× CSE. (**e**) Annexin V-Alexa Fluor 488-gated events were reduced upon SCAL1 overexpression. (**f**) Live cell population (AV−/PI−) increase was accompanied by a decrease of apoptotic cell (AV+) number upon SCAL1 upregulation. (**g**) Annexin V-Alexa Fluor 488-gated events increased upon SCAL1 knockdown. (**h**) SCAL1 knockdown resulted in a reduced live cell population (AV−/PI−) and a greater number of apoptotic (AV+) cells. (**i**,**j**) Fluorescent staining of caspase 3/7 and active mitochondria showed lower caspase 3/7 levels and a higher TMRM intensity in SCAL1-overexpressing cells. (**k**,**l**) SCAL1 knockdown resulted in higher caspase 3/7 staining intensity and lower TMRM levels. Data presented are representative of three independent trials performed in triplicates and are expressed as the mean ± SD. *P < 0.05, **P < 0.01, ***P < 0.001 and ****P < 0.0001.

Resistance to apoptosis was assessed using Annexin V/Propidium Iodide (AV/PI) flow cytometry analysis. Treatment of A549 cells with CSE concentrations of 0 up to 0.5× followed by induction of apoptosis via the addition of menadione sodium bisulfite (MSB) did not result in apoptosis as signified by the high fraction of AV−/PI− and low number of AV+ cells in the population (Fig. 4a,b), suggesting that smoke exposure exhibited minimal to no toxicity. However, in the concentrated CSE setup, a significant shift in the population was observed, with 31.27% AV−/PI− cells, and 66.80% AV+ cells (Fig. 4a,b).

The high number of apoptotic cells may be attributed to an observed increase in caspase 3/7 (Fig. 4c) activation coupled with decreased mitochondrial function (Fig. 4d) in cells exposed to the highest CSE concentration. In CSE-treated A549 in which SCAL1 is overexpressed, cells generally exhibited resistance to apoptotic induction by maintaining low caspase 3/7 activity and preserving mitochondrial integrity (Fig. 4c,d). It is therefore possible that CSE-induced SCAL1 upregulation confers resistance to apoptosis even in the presence of strong inducers.

To further explore the putative anti-apoptotic role of SCAL1 upregulation, SCAL1 overexpression and knockdown were performed in the context of the apoptosis assays used with the various CSE concentrations. Overexpressing SCAL1 in A549 reduced the percentage of AV+ cells from 21.52 to 16.75% (Fig. 4e,f) while SCAL1 knockdown increased these apoptotic cells more than twofold from 6.09 to 13.57% (Fig. 4g,h). This is consistent with the observed decreased caspase 3/7 activity and more intact mitochondria in A549 cells upon overexpression of SCAL1 (Fig. 4i,j). Conversely, SCAL1 knockdown resulted in higher caspase 3/7 levels and lower mitochondrial integrity (Fig. 4k,l).

### SCAL1 detoxifies cells of ROS species

**Figure 5:**
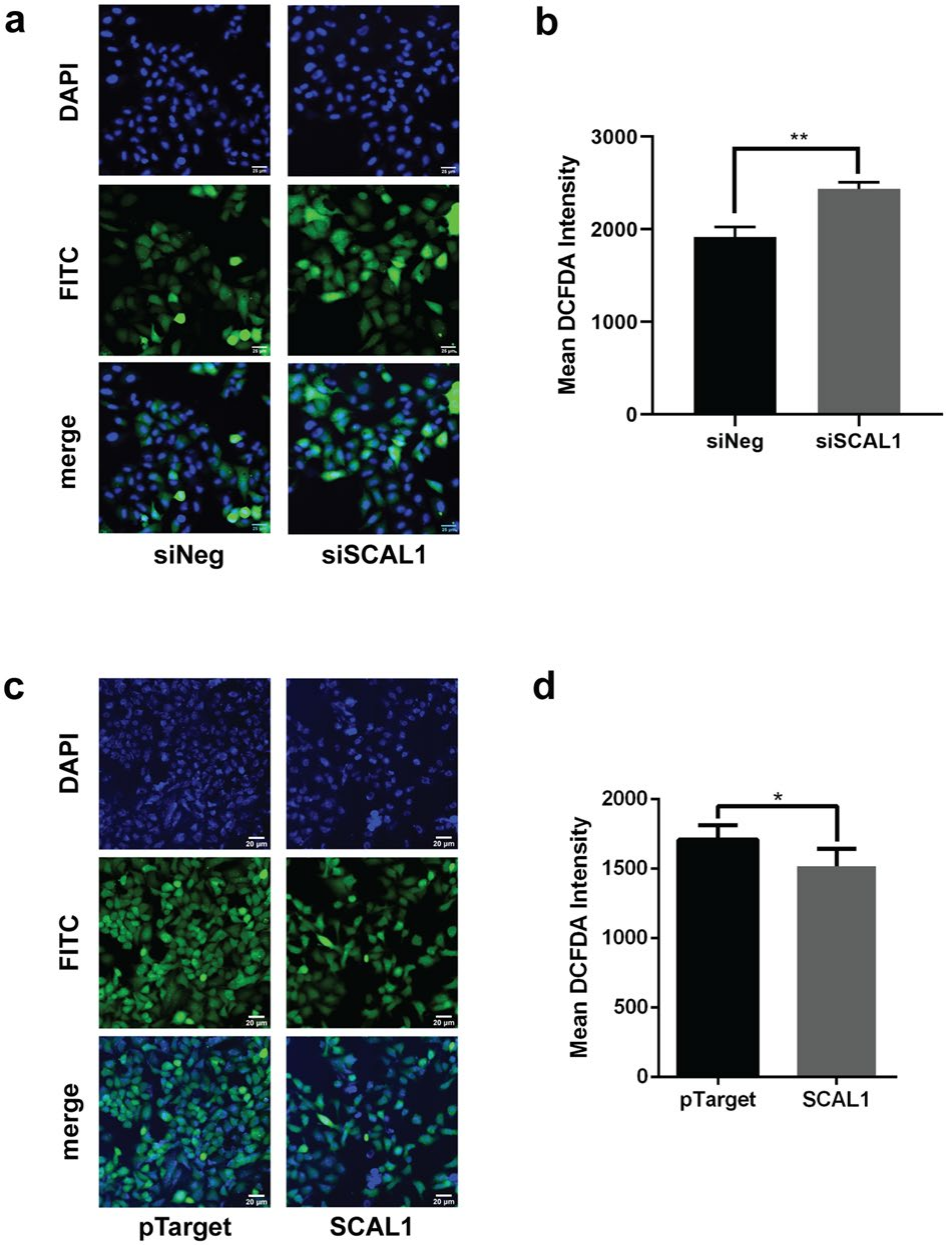
DCFDA staining of CSE-treated, SCAL1-deficient and SCAL1-overexpressing A549 cells to assess production of reactive oxygen species (Olympus IX51, ×100, FITC: 500 ms). (**a**,**b**) The fluorescein-based dye DCFDA exhibits green fluorescence upon oxidation by ROS. The bright green signal observed in A549 cells signifies ROS production. DCFDA intensities normalized against cell count showed increased mean ROS production upon SCAL1 knockdown. (**c**,**d**) DCFDA fluorescence signals were lower in the SCAL1 overexpression setup, suggesting that increased SCAL1 levels decrease ROS production. Data presented are representative of three independent trials performed in triplicates and are expressed as the mean ± SD. *P < 0.05, **P < 0.01, ***P < 0.001 and ****P < 0.0001.

To determine if SCAL1 plays a role in detoxifying cells and reducing ROS species, A549 cells were treated with 0.1× CSE for 24 h to upregulate SCAL1 expression. Cells were then transfected with 30 nM siSCAL1 (Fig. 5a,b) to see if reduced levels of the lncRNA make cells more susceptible to oxidative stress. Intracellular ROS levels were assessed with the cell-permeant DCFDA (2′,7′-dichlorofluorescin diacetate) fluorogenic dye. DCFDA staining of CSE-treated A549 cells revealed an increase in ROS levels upon SCAL1 knockdown, suggesting that cells become more susceptible to oxidative stress when SCAL1 is downregulated.

In contrast, A549 cells transfected with pTargeT-SCAL1 construct yielded lower DCFDA fluorescence levels compared to the vector only control (Fig. 5c,d). This suggests that SCAL1 upregulation consequently leads to reduced intracellular ROS production, and thus the detoxification of A549 lung cancer cells.

These results also lend credence to the posited role of NRF2-mediated upregulation of antioxidant genes such as SCAL1 secondary to cigarette smoke exposure, which protects cells from injury brought about by oxidative stress.

## Discussion

It is generally acknowledged that the acquisition of genetic lesions and formation of DNA adducts, in part due to oxidative damage, drive cigarette smoke-associated lung oncogenesis^32–34^. The mechanisms by which cells survive and remain viable despite these incessant genetic insults, however, remain unclear. The elucidation of the role of NRF2 and how it activates antioxidant genes^35, 36^ as a pro-survival mechanism shed some light on how cells remain viable, albeit not without damage of functional consequence. More recently, lncRNAs were proposed to play a role in detoxification processes as a response to xenobiotic insults.

Upon analysis of global RNA expression in smoke-exposed lung cancer cells, Thai et al.^13^ discovered a novel lncRNA transcript (smoke and cancer-associated lncRNA 1 or SCAL1) that exhibited abnormally high expression levels. They also showed significant elevation of SCAL1 levels in airway epithelia of smokers versus non-smokers, up to 5.3-fold higher in one, and 3.9-fold higher in another study. The present study aimed to further characterize SCAL1 in terms of the oncogenic phenotypes it controls beyond cell viability and proliferation, using A549 adenocarcinomic alveolar epithelial cells as model system. As with other lncRNAs, it remains to be seen whether the observations and results described herein can be extrapolated to other cellular contexts, even within lung tissues, given the high context-specificity of lncRNA function.

CSE and its consequent upregulation of SCAL1 were shown to promote cellular migration. SCAL1 response to CSE exhibited dose-dependence until a specific threshold, beyond which SCAL1 returned to baseline levels (Fig. 1a), quite possibly through the activation of a negative feedback mechanism that effectively inhibited its expression. This trend was also reflected in the migratory capacity of cells exposed to varying concentrations of CSE as shown in Fig. 2a,b. Excessive detoxification may have reduced oxidative electrophilic binding of stress molecules to KEAP1, resulting in its stabilization, and allowed it to bind NRF2 again and facilitate its proteolytic degradation. This, in turn, may switch off nascent transcription of SCAL1 while older SCAL1 transcripts become destabilized.

Enhancement of migratory capacity as well as the extensive cytoskeletal remodeling observed may help provide an explanation for the high metastatic rates in smoke-associated lung carcinoma, as cells become equipped with molecular escape mechanisms that allow them to leave the primary tumor site to establish new tumor colonies elsewhere. The effects of CSE treatment, SCAL1 overexpression, and SCAL1 knockdown on cytoskeletal organization were examined to find a correlation with the observed increase in migratory capacity of A549 lung cancer cells. Cancer cells gain motility and invasive properties by forming matrix-degrading structures such as lamellipodia and filopodia. While lamellipodia are the primary structures for cell locomotion, filopodia occur at high frequencies in cancer cells and are therefore highly correlated with increased invasive capacity^37, 38^. The detection of these actin-rich structures in cells exposed to CSE and upon SCAL1 upregulation, as well as their absence in SCAL1-deficient cells suggest that this lncRNA enhances the migratory capacity of A549 cells.

Treatment with CSE and exogenous SCAL1 overexpression produced overlapping and distinct transient structures associated with migration and invasive capacity, suggesting that in some instances, their effects can be decoupled. Formation of tunneling nanotubes was more readily apparent and extensive in CSE-treated A549 lung cancer cells. These filamentous extensions are under active investigation due to their role in direct cell-to-cell communication and transmission of cellular cargo implicated in tumor invasion and metastasis^39^. Aside from opening discrete communication pathways, TNTs allow for the formation of open-ended channels that mediate membrane continuity to balance stress factors caused by pathological changes and fluctuating conditions such as oxidative stress or nutrient shortage. Intercellular transfer via TNTs thereby gives way to the occurrence of molecular pathways that significantly impact major physiological processes such as cell survival, redox or metabolic homeostasis through ROS detoxification, and mitochondrial heteroplasmy^40, 41^. The formation of TNT-like structures in CSE-exposed cells may be an adaptation mechanism to cope with the oxidative stress caused by smoking. Other transient structures observed in CSE-treated cells include thick peripheral actin rims which are linked to cellular motility as well. They are commonly a positive indicator for the formation of lamellipodia. These actin rims may also play a role in the deformability and malleability of the nucleus during cellular migration^28^.

SCAL1 overexpression in the absence of CSE exposure, on the other hand, revealed transient structures that are also able to cause cytoskeletal disorganization to promote or facilitate cellular migration. Peripheral ruffles, CDRs, perinuclear actin rims, as well as peripherally located and stretched nuclei were prominent. Large multinucleated cells were also observed. Peripheral ruffles and lamellipodia are associated with mesenchymal migration of cancer cells and can initiate both adhesive interactions and extracellular matrix (ECM) remodeling^42^. Peripheral ruffles eventually become CDRs by collapsing inwards, and in so doing are able to disrupt and soften the cytoskeleton, thus enabling migration^43^. CDRs are also known to play a role in micropinocytosis which supports tumor growth^44^. The presence of perinuclear actin rims, peripheral nuclear displacement, and nuclear stretching or deformability all support the cytoskeletal remodeling that needs to happen in preparation for migration and invasion^45^. The transient assembly of a perinuclear actin rim, in particular, is supposed to protect genome integrity until cellular homeostasis is reestablished^46^. The distinct phenotypic effects on cytoskeletal remodeling caused by SCAL1, in the absence of CSE exposure, suggest that SCAL1 upregulation may only partially mediate the oncogenic effects of CSE exposure. Both CSE treatment and SCAL1 overexpression are able to promote migratory capacity.

In CSE concentrations known to upregulate SCAL1 expression, A549 cells were shown to be resistant to apoptosis even in the presence of strong inducers. Overexpression of pTargeT-SCAL1 construct by itself produced similar effects. On the other hand, SCAL1 knockdown resulted in higher caspase 3/7 levels and lower mitochondrial integrity. Taken together, these suggest that SCAL1 upregulation, secondary to cigarette smoke exposure or via alternative mechanisms, does protect cells and promote survival.

Response to oxidative stress was also studied by observing intracellular levels of stress-induced molecules such as ROS. Oxidative damage to DNA as a result of high intracellular ROS levels have been shown to promote cancer-causing mutations^47^. However, tumor cells may also upregulate antioxidant proteins and their regulators for detoxification in order to prevent induction of ROS-mediated apoptosis and ensure cell survival. This was confirmed in the apoptosis assays whereby cigarette smoke-exposed cells that overexpressed SCAL1 showed resistance to induced cell death.

**Figure 6:**
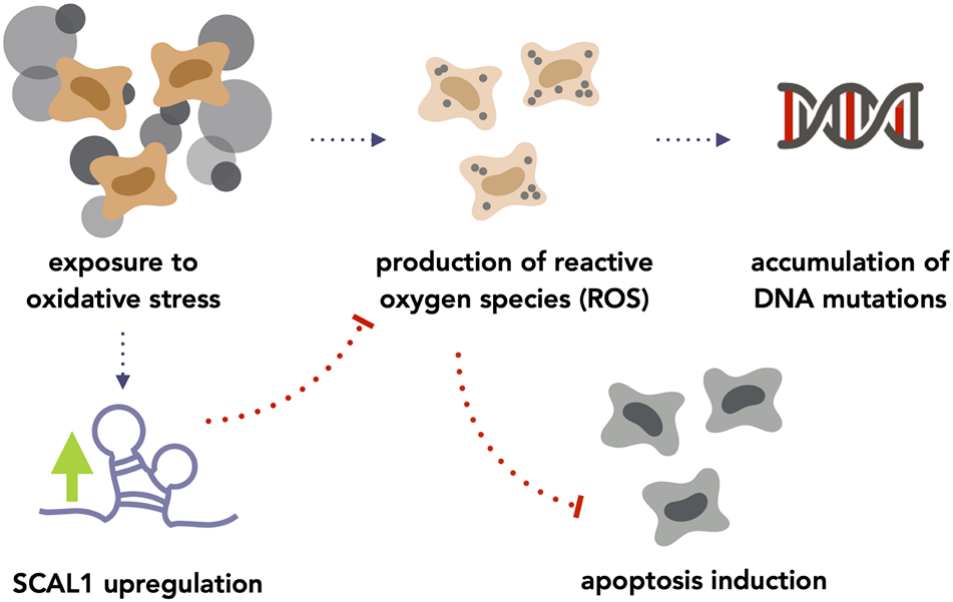
Postulated pathway of SCAL1 upregulation to account for ROS reduction upon CSE exposure. SCAL1 may be upregulated in response to oxidative stress to inhibit ROS production, which may effectively inhibit the induction of apoptosis.

Cigarette smoke exposure of airway epithelial cells has been shown to increase ROS levels^48, 49^. In this study, knockdown and overexpression of SCAL1 were employed to provide proof to earlier suggestions that SCAL1 upregulation may promote detoxification of cells to ensure their viability and survival. Indeed, knockdown of SCAL1 in A549 cells pre-treated with 0.1× CSE to induce SCAL1 upregulation resulted in a corresponding increase in ROS levels. Conversely, overexpression of pTargeT-SCAL1 produced opposite effects. Altogether, these suggest that an increase in SCAL1 expression may lead to a decrease in intracellular ROS and a consequent inhibition of ROS-mediated apoptosis. However, this also implies that cells which have already accumulated possibly deleterious and oncogenic DNA mutations may be spared from programmed cell death and may thus be allowed to further propagate. In this case, oxidative stress may force cells to adapt by overexpressing antioxidant genes such as SCAL1 to resist cell death, a negative implication of which is that damaged and tumorigenic cells are effectively protected and allowed to survive (Fig. 6).

It is firmly established that chronic and not acute cigarette smoke exposure induces carcinogenesis. However, in vitro experiments following acute exposure, such as those described in this study, are instructive in that they show how cellular oncogenic phenotypes are promoted and may become irreversible when exposure to cigarette smoke is sustained, and DNA and cellular damage are allowed to override repair mechanisms. The fact that SCAL1 was discovered from analysis of dysregulated transcripts in lung cancer patients with a history of smoking suggests that chronic exposure to cigarette smoke can sustain or stabilize SCAL1 expression in vivo.

The results described in this study suggest a protective role for SCAL1 in the context of cigarette smoking, with the caveat that cells with damaged DNA are also allowed to survive and multiply. The oncogenic effects of SCAL1 can also be decoupled from those of cigarette smoke, suggesting that this lncRNA may be upregulated via other mechanisms to promote oncogenicity. Either way, its potential as a diagnostic and prognostic biomarker is already being considered. Sun et al.^50^, in their study involving 68 patients, showed that there is a statistically significant association between increased SCAL1 levels and larger tumor size as well as advanced tumor-node-metastasis (TNM) stage. Further, they showed that increased SCAL1 expression is associated with poor prognosis and decreased overall survival. Targeting SCAL1 may also be a viable therapeutic option. SCAL1 knockdown may sensitize cancer cells to targeted therapies by allowing anti-proliferative, anti-migratory and cell death signals to put the brakes on tumor growth, metastatic spread and resistance to apoptosis.

## Materials and methods

### Culture of A549 cells in maintenance media and CSE-infused media

Adenocarcinomic alveolar epithelial cells (A549; ATCC; Manassas, VA, USA) were cultured and maintained in T-25 culture flasks with Dulbecco’s Modified Eagle Medium (DMEM; Gibco^®^, Thermo Fisher Scientific, Inc., Waltham, MA, USA) and 10% fetal bovine serum (FBS; Gibco^®^) [DMEM + 10%FBS] in controlled environmental conditions (37 °C, 5% CO_2_). Modified culture media containing CSE was formulated by attaching a cigarette stick to the nozzle of a 50 ml sterile syringe containing 10 ml of DMEM + 10%FBS. After the cigarette stick was lit, mainstream smoke was suctioned by repeated pushing and pulling of the plunger. Upon consuming the stick, the media within the syringe was shaken vigorously for 20 s to ensure maximum dissolution of the smoke. This procedure was repeated 5 times, after which the resulting medium was sterilized through a 0.22 µm Minisart^®^ filter and was designated as concentrated CSE-DMEM (1 cigarette stick: 10 ml DMEM + 10% FBS).

A549 cells were seeded on assay plates and incubated for approximately 24 h to allow attachment to the substrate. Subsequently, the medium was replaced with CSE-DMEM of different dilutions [concentrated CSE (1×), twofold dilution (0.5×), tenfold dilution (0.1×), 50-fold dilution (0.02×), and 100-fold dilution (0.01×)] while one well was replenished with maintenance medium as a control. A549 cells were cultured for an additional 48 h before performing downstream functional assays and analyses.

### RNA extraction

The RNEasy Plus Mini Kit (Qiagen Sciences, Inc., Germantown, MD, USA) was used to extract total RNA from A549 seeded at a density of 150,000 cells per well on a 12-well plate. RNA concentration was determined spectrophotometrically (λmax = 260 nm) using the Nanodrop 2000c spectrophotometer (Thermo Fisher Scientific) and samples were stored at − 80 °C until further use.

### Detection of SCAL1 expression levels via RT-qPCR

RT-qPCR was performed using the PowerUp™ SYBR Green Real-Time PCR (Thermo Fisher Scientific) kit according to the manufacturer’s recommended protocol. Using 200 U of M-MLV reverse transcriptase (Promega^®^, Madison, WI, USA), 2000 ng of RNA was reverse transcribed into cDNA. An annealing reaction mixture containing oligo-dTs, random hexamers, and the RNA sample was incubated at 70 °C for 5 min. The M-MLV reverse transcriptase buffer was then added to the mixture to a final 1× concentration. In addition, 25 U of RNase inhibitor (Promega^®^), 0.075 mM of deoxynucleoside triphosphate (dNTPs), and 200 U of M-MLV reverse transcriptase were included in the reaction mixture to a final volume of 25 µl. The reverse transcription process was facilitated by an hour-long incubation at 37 °C and the resulting cDNA was stored at − 20 °C until use.

SCAL1 transcript levels in A549 cells exposed to different titers of CSE-DMEM were compared. To ascertain the effect of recovery after acute exposure to CSE on SCAL1 expression, A549 cells were treated with 0.1× CSE-DMEM for 48 h and were then cultured in CSE-free DMEM for 24 h. Table 1 shows the primers used to amplify the SCAL1 transcript and the cyclophilin gene as a normalization control.

**Table 1:** Primers used in reverse transcription-quantitative PCR (RT-qPCR).

Designation	Sequence (5′ to 3′)
*SCAL1 (qPCR)*	Forward: CTGTCCCTCAGTGTTCTACTT C
	Reverse: CTGGCATCCATTGTGTCTTATTT
*Cyclophilin*	Forward: CCT AAA GCA TAC GGG TCC TGG CAT C
	Reverse: GTG GAG GGG TGC TCT CCT GAG CTA C

### Generation of SCAL1 lncRNA gene expression construct

SCAL1 gene blocks were obtained from Integrated DNA Technologies (Coralville, IA, USA) (ref.no. 98409609). This transcript was cloned into the pGEM^®^-T Easy cloning vector (Promega^®^) via non-directional TA-cloning. A-overhangs were added prior to ligation as follows: a tube containing 1× Titanium Taq buffer, 5× Titanium Taq polymerase (Clontech Laboratories, Inc., Mountain View, CA, USA), and 1 mM dNTPs was first incubated at 95 °C for 5 min, after which 24.5 ng of the gene block was added and the resulting mixture was incubated at 72 °C for 15 min. From the solution with poly A-appended SCAL1 blocks, 1.1 µl was added to a separate mixture of 2× T4 DNA ligase buffer (Roche^®^ Diagnostic, GmbH, Mannheim, Germany), 50 ng of pGEM^®^-T Easy cloning vector (Promega^®^), and 0.25 U of T4 DNA ligase buffer (Roche^®^). The 5.2-µl solution was then incubated with the following profile: 4 °C for 2 h, 22 °C for 4 h, and 14 °C overnight after which it was used for transformation into DH5α ultracompetent E. coli cells. Recombinant pGEM^®^-T Easy-SCAL1 constructs with verified sequences were digested with EcoRI, subcloned into the pTargeT™ mammalian expression vector, and verified by sequencing for accuracy and directionality.

### Knockdown of SCAL1 in A549 cells

In a 12-well plate, approximately 300,000 A549 cells were seeded per well and transfected with 30 nM of the AllStars (Qiagen Sciences, Inc., Germantown, MD, USA) negative control siRNA (si-NEG, cat.no. 1027280) or 30 nM of SCAL1-siRNA (Hs_LOC100505994_3; Qiagen FlexiTube siRNA SI05713505). The negative control used is a non-silencing control siRNA with no known homology to any mammalian gene. Transfecting a universal siRNA control accounts for the stress of transfection and was used as the basis for determining normal, basal levels of gene expression.

In all setups, A549 cells were transfected with the appropriate amounts of siRNA with Lipofectamine™ 3000 transfection reagent (Invitrogen). RNA-Lipofectamine™ complexes were prepared as follows: per transfection reaction, the 30 nM of siRNA was diluted to a final volume of 50 µl with Opti-MEM. In another tube, 3 µl of the Lipofectamine™ 3000 transfection reagent was diluted to 50 µl with Opti-MEM. The RNA and Lipofectamine™ solutions were then mixed and incubated at room temperature for 10–15 min to allow for complexation to occur, after which the resulting 100 µl solution was added to adherent A549 cells. Forty-eight hours post-transfection, the cells were harvested for subsequent molecular, morphological, and functional characterization experiments. Efficiency of SCAL1 knockdown was assessed via RT-qPCR as previously described.

### Transfection of pTargeT™-SCAL1 construct into A549 cells

Twenty-four hours after seeding 150,000 A549 cells into a 12-well plate, 1000 ng of the construct was transfected using the same method employed for lipofection of SCAL1-siRNA in knockdown studies. In separate wells, 1000 ng of empty pTargeT™ vector and empty pmR-ZsGreen1 vector were transfected as negative control and transfection efficiency tracker, respectively.

For each setup, cell count was determined using the Bio-Rad TC20™ Automated Cell Counter (Bio-Rad Laboratories, Inc., Hercules, CA, USA) from which the appropriate number of cells to seed per assay was calculated. All assays (minimum of three trials) were performed with samples seeded in triplicates to ensure repeatability of obtained results.

### Scratch wound assay

In the scratch wound assay, approximately 20,000 A549 cells were seeded per well in a 96-well clear-bottom black plate (Corning) with DMEM + 10%FBS maintenance medium. Cells were incubated at 37 °C with 5% CO_2_, to allow cells to attach. In order to facilitate quantification of wound closure rate, cells were stained with the fluorescent dye calcein. Briefly, Calcein AM was added to each well at a final concentration of 2 µg/ml in low serum medium (DMEM + 2%FBS). A sterile toothpick was then used to scratch the well in a straight line to create a wound in the confluent cell monolayer. Wells were washed with 1× D-PBS and subsequently imaged using the GE IN Cell Analyzer 6000 high-content imaging system (GE Healthcare Life Sciences; Marlborough, Massachusetts, USA). Cells were again stained for calcein after 16 h and imaged afterwards. Wound closure rates were measured with GE IN Cell Analyzer 6000 Analysis Software.

### Monitoring changes in cytoskeletal architecture

Analysis of cellular morphology was facilitated by staining actin filaments with fluorescein isothiocyanate (FITC) conjugated phalloidin (5 µg/ml; Life Technologies, Thermo Fisher Scientific, Inc., Waltham, MA, USA) and nuclei with Hoechst 33342 (5 µg/ml; Life Technologies). A549 cells seeded on a 96-well clear-bottom black plate (Corning) (5000 cells). Twenty-four hours after seeding, cells were either treated with CSE or transfected with the siRNA or the overexpression plasmid. After 48 h, culture media was removed, and wells were washed with 1× PBS before adding 4% paraformaldehyde for sample fixation at room temperature for 1 h. Cells were then washed with ice-cold PBS three times for 5 min each, after which 0.1% Triton™ X-100 (Sigma-Aldrich, St. Louis, MO, USA) was added to permeabilize the cells at room temperature for 10 min. This was followed by a blocking step which involved the addition of 1% BSA and incubation at room temperature for 1 h with constant agitation (60 rpm). After washing cells with ice-cold 1× PBS, FITC-conjugated phalloidin was added and samples were incubated with the fluorescent cytoskeletal stain for 30 min with constant agitation (60 rpm) at room temperature in the dark. Cells were again washed with ice-cold 1× PBS, followed by the addition of the nuclear stain Hoechst 33342 and subsequent incubation for 15 min at room temperature in the dark. After a final wash step with ice-cold 1× PBS, fluorescent images were obtained with the GE IN Cell Analyzer 6000 high-content imager and the Olympus IX3-HOS microscope (Olympus Corp., Tokyo, Japan) using the green fluorescent filter (λex/λem:490/525 nm) to visualize stained filamentous actin structures, and the blue fluorescent filter (λex/λem:355/465 nm) to visualize the nuclei.

### Staining of reactive oxygen species with DCFDA

Visualization and measurement of intracellular levels of reactive oxygen species (ROS) was performed by staining with 2′,7′-dichlorofluorescein diacetate (DCFDA; Sigma-Aldrich, St. Louis, MO, USA). Reseeding for DCFDA staining was done by placing 8000 A549 cells per well on a 96-well clear-bottom black plate (Corning). For SCAL1 knockdown, cells were treated with 0.1× CSE for 24 h to induce SCAL1 upregulation and was subsequently transfected with 30 nM siSCAL1 to reduce intracellular SCAL1. For SCAL1 overexpression, cells were transfected with pTargeT-SCAL1 and incubated for 24 h before treating with 0.1× CSE. Forty-eight hours post-transfection of either siSCAL1 or pTarget-SCAL1, the DCFDA stain (50 µM) was added to each well. The assay plate was incubated at 37 °C for 20 min before observation and imaging with the GE IN Cell Analyzer 6000 high-content imager at λex/λem:485/535 nm.

### Annexin V/propidium iodide flow cytometry analysis

In a Corning flat-bottom polystyrene 12-well plate, 150,000 cells were seeded and allowed to attach. Apoptosis was induced by incubation with 100 µM menadione sodium bisulfite (Sigma-Aldrich) in reduced serum medium (DMEM + 4%FBS) for 12 h. All suspended and adherent cells were collected, after which apoptotic cells were observed using the Alexa Fluor 488 Annexin V/Dead Cell Apoptosis Kit (Thermo Fisher Scientific) according to the manufacturer’s instructions.

### Measurement of Caspase 3/7 levels and mitochondrial integrity

A549 cells were seeded in a Corning flat-bottom polystyrene 96-well plate (5000 cells). Subsequently, 24 h after seeding, apoptosis was induced by incubation with 100 µM menadione sodium bisulfite (Sigma-Aldrich) in reduced serum medium (DMEM + 4%FBS) for 12 h. Caspase 3/7 activity and mitochondrial activity were detected by incubation with 2 µM of CellEvent™ Caspase-3/7 Green Detection Reagent (Thermo Fisher Scientific) and 2 µM of Image-iT™ TMRM Reagent (Thermo Fisher Scientific) for 30 min. Fluorescent images were obtained with the GE IN Cell Analyzer 6000 high-content imager (Caspase-3/7 λex/λem:503/530 nm; TMRM λex/λem:548/574 nm). Quantification of fluorescent signals was performed using the IN Cell Developer Toolbox v1.6 (GE Healthcare).

### Statistical analysis

To quantify the differences between the control and various experimental setups, the unpaired two-tailed Student’s t test was used. The quantifications in the various figures are presented as the mean ± standard deviation (SD). P < 0.05 was used to define statistical significance.

### Ethical approval

This article does not contain any studies with human participants or animals performed by any of the authors.

## Supplementary Information

Supplementary Information 1.

## Data Availability

Data generated in this study are available from the corresponding author on reasonable request.
