# Supplementary material for: Concomitant and decoupled effects of cigarette smoke and SCAL1 upregulation on oncogenic phenotypes and ROS detoxification in lung adenocarcinoma cells
Source: Sci Rep. 2021 Sep 15;11:18345. doi: 10.1038/s41598-021-97869-1 (PMC8443756; doi:10.1038/s41598-021-97869-1)
Supplement: Supplementary file 1 — Supplementary Information 1. [file 41598_2021_97869_MOESM1_ESM.pdf]

Concomitant and decoupled effects of cigarette smoke and SCAL1 upregulation on oncogenic phenotypes and ROS detoxification in lung adenocarcinoma cells

Carmela Rieline V. Cruz [1,2,§], Jose Lorenzo M. Ferrer [1,§] and Reynaldo L. Garcia [1,*]

1 Disease Molecular Biology and Epigenetics Laboratory, National Institute of Molecular Biology and Biotechnology, University of the Philippines Diliman, Quezon City 1101, Philippines; c.cruz@stud.uni-goettingen.de (C.R.V.C.); jmferrer2@up.edu.ph (J.L.M.F.)

2 Department of Developmental Biology, International Max Planck Research School, Justus-von-Liebig-Weg 11, 37077 Göttingen, Germany

§ These authors contributed equally to this work.

* Correspondence: reygarcia@mbb.upd.edu.ph; Tel.: +63 2 9818500 Loc. 3953

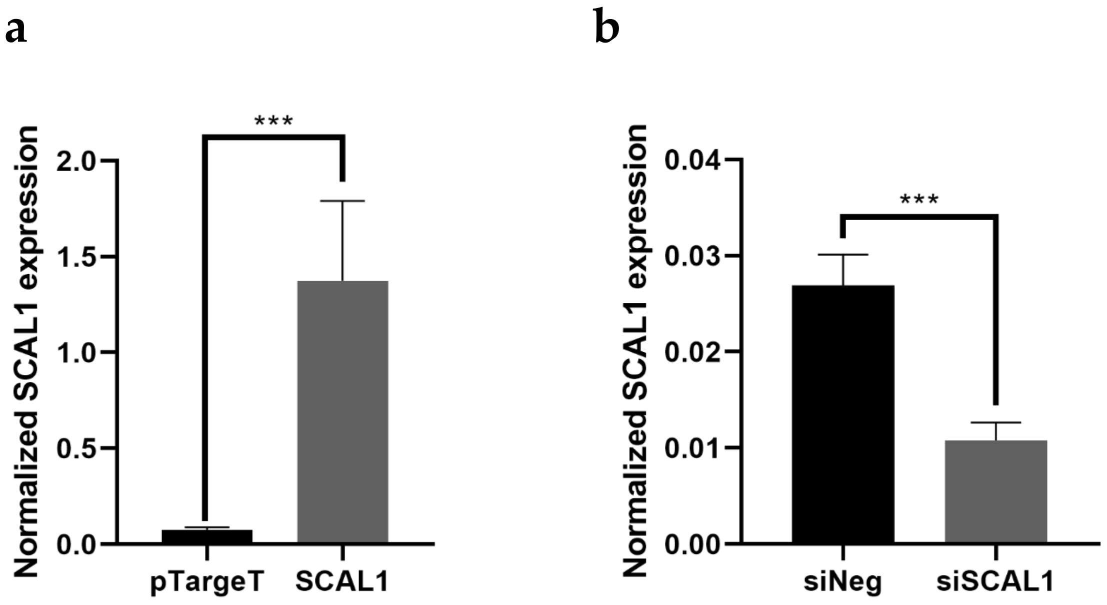

Supplementary Figure S1. Overexpression and knockdown of SCAL1 in transfected A549 cells. Representative experiments showing (a) overexpression and (b) knockdown of SCAL1 in A549 cells upon transfection with the pTargeT-SCAL1 plasmid and SCAL1-specific siRNA (30 nM) compared with their respective controls.
